# Supplementary material for: Copolymerization of Phthalic Anhydride with Epoxides Catalyzed by Amine-Bis(Phenolate) Chromium(III) Complexes
Source: Polymers (Basel). 2021 May 28;13(11):1785. doi: 10.3390/polym13111785 (PMC8197922; doi:10.3390/polym13111785)
Supplement: Supplementary file 1 [file polymers-13-01785-s001.zip › polymers-1231387-supplementary.pdf]

polymers

MDPI

Supplementary Material: Copolymerization of Phthalic Anhydride with Epoxides Catalyzed by Amine-bis(phenolate) Chromium(III) Complexes

K. Bester, A. Bukowska, A. Sobota, M. Pytel, W. Bukowski

1. General information

All works with air and/or water-sensitive compounds were carried out under dry argon atmosphere in a glove box or using the standard Schlenk techniques.

1.1. Materials

Cyclohexene oxide (Sigma-Aldrich, 98%), 4-vinyl-1-cyclohexene 1,2-epoxide (Sigma-Aldrich, 98%), propylene oxide (Fluka, 97%), 1,2-butylene oxide (Fluka, 99%), styrene oxide (Fluka, 97%), epichlorohydrin (Sigma-Aldrich, 99%), phenyl glycidyl ether (Sigma-Aldrich, 99%) and pentane were stirred with calcium hydride (Sigma-Aldrich, 90%) under argon for 2 days, vacuum transferred to hot-gun dried Schlenk flasks, degassed and stored under argon until use. Phthalic anhydride (Sigma-Aldrich, 99%) was refluxed with chloroform (10 g anhydride in 40 ml chloroform) under argon for 1 h followed by hot filtration. The solvent was evaporated from the filtrate using a rotary evaporator, and the resulting white solid was washed with diethyl ether, filtered off, and dried under vacuum for 12 h. Next, the freshly recrystallized anhydride was resublimed and stored under argon in the glove box until use. Tetrahydrofuran and toluene were refluxed with sodium/benzophenone under argon until the deep blue colour of benzophenone ketyl was formed. The dry solvents were collected directly prior to use. 2-*tert*-Butyl-4-fluorophenol was prepared according to the literature procedure.[S1] All other chemicals and reagents were purchased from commercial suppliers (Sigma-Aldrich, Alfa Aesar, Acros) and used as received.

1.2. Methods

NMR experiments. ^{1}H and ^{13}C NMR spectra were recorded at 298 K temperature in $CDCl_3$ solutions using a Bruker Avance 500 MHz spectrometer (^{1}H, 500 MHz and ^{13}C, 125 MHz). TMS was used as a standard. Samples for analysis were prepared by dissolving 15-30 mg low molecular or 60-80 mg polymeric products in *ca.* 0.6 ml of $CDCl_3$.

Infrared experiments. FT-IR spectra were recorded using a Thermo Scientific Nicolet 8700 spectrometer in the range of 4000-650 cm^{-1}. All measurements were performed by the ATR method.

Mass spectroscopy. Laser desorption/ionization (LDI) time-of-flight (ToF) mass spectrometry experiments were performed using a Bruker Autoflex Speed reflectron time-of-flight mass spectrometer equipped with a SmartBeam II laser (352 nm) in the range of 80-2000 m/z. Laser impulse energy and the rate of laser repetition were approximately 60–120 μJ and 1000 Hz, respectively. The first accelerating voltage was held at 19 kV and the second ion source voltage at 16.7 kV. The reflector voltages applied were 21 kV (first) and 9.55 kV (second). The analytical data were collected and analysed using the software provided with the Autoflex instrument (FlexAnalysis version 3.3). Mass calibration (typically cubic calibration based on five to seven points) was performed using internal standards (gold ions and clusters from Au^{+} to $Au10^{+}$ depending on the m/z range). The sum of *ca.* 7000 scans was collected for each sample. Analytical samples were prepared by dissolution/suspending *ca.* 0.5 mg substances analysed in 1 mL toluene. One drop of the resulting solution/suspension was placed on AuNPET.[S2]

Gel permeation chromatography (GPC). Molecular weight determinations (M_n and M_w) were conducted using an Agilent 1100 HPLC instrument equipped with a degasser, a thermostat, a RI detector, and a MIXED-D 300 x 7.5 mm column. The analytical data were collected and analysed using the software provided with the instrument (ChemStation for LC Rev. A.10.02 with ChemStation GPC Data Analysis Software Rev. A.02.02). Polystyrene standards (Polymer Laboratories, M_p = 580, 1230, 3090, 4750, 9570, 19500, 27810, 67600, 117700, 301600 g/mol) were used to calibrate the system. All analyses were performed at 35°C using THF as an eluent at a flow rate of 0.5 mL/min. The concentrations of the analysed samples were about 0.5 %. The samples were filtered through 0.2 μm PTFE filters before their injection. The injection volume was 20 μL.

Differential scanning calorimetry (DSC). DSC measurements were carried out using a Mettler-Toledo DSC-1 instrument. The glass transition temperatures (T_g) of polymers were determined from the second heating at the heating rate of 10 °C/min, from -50 to +200 °C, under nitrogen atmosphere.

ICP-OES analysis. The content of chromium in the synthesized metal complexes were determined using an ICP-OES ULTIMA 2 HORIBA JOBIN YVON spectrometer (λ = 283.563 nm, photomultiplier voltage of 950 V and generator power 1400 W). The samples of the complexes (about 20 mg) were mineralized in conc. nitric acid (5.0 ml) using a *Plazmatronika Uni Claver II* microwave mineralizer and diluted with demineralized water before their analysis.

Melting point measurements. The melting points of the obtained ligands were determined using an SRS OptiMelt MPA100 instrument with the heating rate of 2°C/min.

2. Syntheses

2.1. Synthesis of amine-bis(phenolate) ligands

Amine-bis(phenolate) ligands **1-8** were prepared using water as a reaction medium. Three 2,4-disubstitiuted phenols differing in substituents at position 5, three different amines, and formaldehyde were used as reactants (*Scheme S1*). The procedure described in the literature with minor changes was applied.[S3-S8]

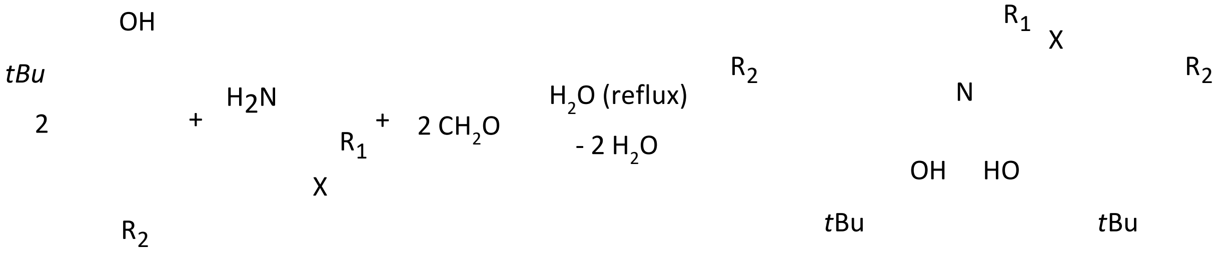

ligand	R_1-X	R_2
1	CH_2NMe_2	*t*Bu
2		OMe
3		F
4	CH_2OMe	*t*Bu
5		OMe
6		F
7	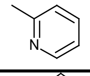	*t*Bu
8	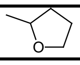	*t*Bu

Scheme S1. Synthesis of amine-bis(phenols) **1-8**.

General procedure. The proper phenol (40 mmol), formaldehyde (44 mmol, as 37% formalin), and water (35 ml) were placed in a round bottom flask fitted with a magnetic stir bar. The resulting suspension was heated to 100°C stirring constantly and then the

proper amine (20 mmol) was added. The reaction mixture was refluxed for 24 h and then cooled to room temperature. The water phase was next decanted and the remaining solid suspended in cold methanol (20 ml). The final product was isolated by filtration and purified by recrystallization from CH_2Cl_2/CH_3OH mixture.

(1): Yield 6.32 g, 60.3 %, white solid, mp = 153,0-155,0°C; **^{1}H-NMR** ($CDCl_3$) δ ppm: 8.79 (br. s, 2H); 7,24 (d, *J*=2Hz, 2H); 6.86 (d, *J*=2Hz, 2H); 4.30-4.20 (m, 1H), 4.04-3.98 (m, 1H); 3.90-3.85 (m, 1H); 3.78-3.73 (m, 4H); 2.63-2.57 (m, 1H); 2.53-2.48 (m, 1H); 1.95-1.85 (m, 3H); 1.58-1.49 (m, 1H), 1.40 (s, 18H); 1.26 (s, 18H). **^{13}C-NMR** ($CDCl_3$) δ ppm: 153.0; 140.6; 136.0; 125.0; 123.4; 121.4; 77.5; 68.3; 57.5; 55.9; 35.0; 34.1; 31.7; 29.6; 29.6; 25.2.; **HRMS** (AuNPET LDI-ToF) m/z: *calculated for* $C_{34}H_{57}N_2O_2^+$ $[M+H]^+$ = 525.4415 *found* 525.4370, *for* $C_{34}H_{56}N_2NaO_2^+$ $[M+Na]^+$ = 547.4228 *found* 547.4238. **FT-IR** (ATR diamond, cm^{-1}): 2955.13, 2803.99, 1479.86, 1441.53, 1415.49, 1361.50, 1300.75, 1216.38, 1197.82, 1165.03, 1109.11, 875.52, 784.16.

(2): Yield 7.43 g, 79,0 %, white solid, mp = 149,5-149,9°C; **^{1}H-NMR** ($CDCl_3$) δ ppm: 9.39 (s, 2H); 6.79 (d, $^4J_{H\text{-}H}$ = *3 Hz*, 2H); 6.46 (d, $^4J_{H\text{-}H}$ = *3 Hz*, 2H); 3.72 (s, 6H); 3.56 (s, 4H); 9.39 (s, 2H); 2.60-2.49 (m, 4H); 2.26 (s, 6H); 1.37 (s, 18H). **^{13}C-NMR** ($CDCl_3$) δ ppm: 151.4; 149.6; 138.4; 122.9; 113.2; 112.5; 56.2; 55.6; 49.0; 44.6; 35.0; 29.3. **HRMS** (AuNPET LDI-ToF) m/z: *calculated for* $C_{28}H_{44}N_2NaO_4^+$ $[M+Na]^+$ = 495.3188 *found* 495.3108. **FT-IR** (ATR diamond, cm^{-1}): 2949.83, 1480.10, 1361.98, 1283.64, 1220.48, 1200.71, 1162.14, 1106.94, 1042.58, 982.55, 923.74, 878.90, 811.16, 780.31.

(3) Yield 6.97 g, 77,0 %, white solid, mp = 177.1-179.8°C; **^{1}H-NMR** ($CDCl_3$) δ ppm: 9.68 (bs, 2H); 6.91 (dd, $^3J_{H\text{-}F}$ = *11 Hz*, $^4J_{H\text{-}H}$ = 3 Hz, 2H); 6.62 (dd, $^3J_{H\text{-}F}$ = *8 Hz*, $^4J_{H\text{-}H}$ = 3 Hz, 2H); 3.55 (s, 4H); 2.60-2.52 (m, 4H); 2.28 (s, 6H); 1.36 (s, 18H).**^{13}C-NMR** ($CDCl_3$) δ ppm: 155.5 (d, $^1J_{H\text{-}F}$ = *234 Hz*); 151.5 (d, $^4J_{H\text{-}F}$ = *1 Hz*), 139.0 (d, $^3J_{H\text{-}F}$ = *6 Hz*); 123.1 (d, $^3J_{H\text{-}F}$ = *6 Hz*); 113.9 (d, $^2J_{H\text{-}F}$ = *22 Hz*); 113.5 (d, $^2J_{H\text{-}F}$ = *24 Hz*); 55.7; 55.6; 49.0; 44.6; 35.0; 29.2. **HRMS** (AuNPET LDI-ToF) m/z: *calculated for* $C_{26}H_{38}F_2N_2NaO_2^+$ $[M+Na]^+$ = 471.2788 *found* 471.2682.

FT-IR (ATR diamond, cm^{-1}): 2952.48, 1604.73, 1431.89, 1361.98, 1292.31, 1292.31, 1204.09, 1121.89, 1025.22, 969.78, 935.31, 878.42, 867.09, 859.37, 812.37, 777.65, 761.50.

(4) Yield 7.73 g, 75.6 %, white solid, mp = 132.5-134.5 °C; **^{1}H-NMR** ($CDCl_3$) δ ppm: 8.47 (br. s, 2H); 7.20 (d, *J*=2Hz, 2H); 6.87 (d, *J*=2Hz, 2H,); 3.73 (s, 4H); 3.54 (t, *J*=5Hz, 2H); 3.46 (s, 3H); 2.74 (t, *J*=5Hz, 2H); 1.40 (s, 18H); 1.26 (s, 18H). **^{13}C-NMR** ($CDCl_3$) δ ppm: 152.9; 140.8; 136.0; 124.9; 123.4; 121.6; 71.4; 58.8; 58.0; 51.3; 35.0; 34.1; 31.6; 29.6. **HRMS** (AuNPET LDI-ToF) m/z: *calculated for* $C_{33}H_{54}NO_3^+$ $[M+H]^+$ = 512.4098 *found* 512.4065, *for* $C_{33}H_{53}NNaO_3^+$ $[M+Na]^+$ = 534.3912 *found* 534.3901. **FT-IR** (ATR diamond, cm^{-1}): 3364.60, 2956.58, 1481.54, 1417.18, 1358.85, 1221.92, 1122.37, 1097.06, 1070.78, 1015.58, 992.68, 878.18, 799.83, 725.34.

(5) Yield 5.58 g, 60.7 %, white solid, mp = 140.6-142.9 °C; **^{1}H-NMR** ($CDCl_3$) δ ppm: 8.15 (br. s, 2H); 6.80 (d, *J*=3 Hz, 2H); 6.46 (d, *J*=3 Hz, 2H,); 3.72 (s, 6H); 3.71 (s, 4H); 3.49 (t, *J*=5 Hz, 2H); 3.44 (s, 3H); 2.72 (t, *J*=5 Hz, 2H); 1.39 (s, 18H). **^{13}C-NMR** ($CDCl_3$) δ ppm: 151.8; 149.2; 138.4; 122.9; 113.3; 112.4; 71.3; 58.8; 57.7; 55.6; 51.4; 34.9; 29.4. **HRMS** (AuNPET LDI-ToF) m/z: *calculated for* $C_{27}H_{41}NO_5^+$ $[M]^+$ = 459.2979 *found* 459.2991, *for* $C_{27}H_{41}NNaO_5^+$ $[M+Na]^+$ = 482.2872 *found* 482.2889. **FT-IR** (ATR diamond, cm^{-1}): 3347.30, 2949.35, 1603.52, 1475.04, 1431.89, 1360.77, 1316.18, 1271.82, 1216.38, 1134.66, 1063.31, 997.26, 956.76, 866.36, 838.40, 765.36.

(6) Yield 4.13 g, 47 %, white solid, mp = 108.8-110.4 °C; **^{1}H-NMR** ($CDCl_3$) δ ppm: 8.35 (bs, 2H); 6.92 (dd, $^3J_{H\text{-}F}$ = *11 Hz*, $^4J_{H\text{-}H}$ = 3 Hz, 2H); 6.62 (dd, $^3J_{H\text{-}F}$ = *8 Hz*, $^4J_{H\text{-}H}$ = 3 Hz, 2H); 3.69 (s, 4H); 3.50 (t, $^3J_{H\text{-}H}$ = *5 Hz*, 2H); 3.46 (s, 3H); 2.72 (t, $^3J_{H\text{-}H}$ = *5 Hz*, 2H); 1.38 (s, 18H), **^{13}C-NMR** ($CDCl_3$) δ ppm: 155.7 (d, $^1J_{H\text{-}F}$ = *234 Hz*); 151.1 (d, $^4J_{H\text{-}F}$ = *3 Hz*), 139.0 (d, $^3J_{H\text{-}F}$ = *5 Hz*); 123.1 (d, $^3J_{H\text{-}F}$ = *6 Hz*); 113.9 (d, $^2J_{H\text{-}F}$ = *25 Hz*); 113.5 (d, $^2J_{H\text{-}F}$ = *24 Hz*); 71.4; 58.9; 57.4; 51.5; 35.0; 29,2. **HRMS** (AuNPET LDI-ToF) m/z: *calculated for* $C_{25}H_{36}F_2NO_3$ $[M+H]^+$ = 436.2658 *found* 436.2550, *for* $C_{25}H_{35}F_2NNaO_3$ $[M+Na]^+$ = 458.2472 *found* 458.2398. **FT-IR** (ATR diamond, cm^{-1}): 3356.01, 2953.45, 2866.67, 1480.10, 1359.09, 1240.49, 1217.10, 1041.13, 798.87, 758.37, 724.86, 650.13.

(7) Yield 9.51 g, 87.3%, white solid, mp = 188.9-193.9 °C; **^{1}H-NMR** ($CDCl_3$) δ ppm: 10.55 (br. s, 2H); 8.71-8.69 (m, 1H); 7.75-7.65 (m, 1H); 7.30-7.25 (m, 1H); 7.20 (d, *J=2Hz,* 2H); 7.13-7.08 (m, 1H);); 6.92 (d, *J=2Hz,* 2H); 3.88-3.80 (m, 6H); 1.40 (s, 18H); 1.26 (s, 18H). **^{13}C-NMR** ($CDCl_3$) δ ppm: 157.6; 154.6; 148.8; 141.1; 138.7; 136.4; 126.1; 125.6; 124.4; 123.9; 122.6; 57.6; 56.3; 35.6; 34.7; 32.0; 30.0. **HRMS** (AuNPET LDI-ToF) m/z: *calculated for* $C_{36}H_{53}N_2O_2^+$ $[M+H]^+$ = 545.4101 *found* 545.4088, *for* $C_{36}H_{52}N_2NaO_2^+$ $[M+Na]^+$ = 567.3915 *found* 567.3888. **FT-IR** (ATR diamond, cm^{-1}): 3089.27, 2949.83, 1597.49, 1480.34, 1433.33, 1361.26, 1293.04, 1230.12, 1203.36, 1162.87, 1122.61, 1094.16, 1003.28, 974.84, 875.28, 862.02, 758.85, 685.09.

(8) Yield 9.73 g, 79.1%, white solid, mp = 156.5-161.6°C; **^{1}H-NMR** ($CDCl_3$) δ ppm: 8.79 (br. s, 2H); 7.24 (d, *J*=2 Hz, 2H); 6.86 (d, *J*=2Hz, 2H); 4.30-4.20 (m, 1H), 4.04-3.98 (m, 1H); 3.90-3.85 (m, 1H); 3.78-3.73 (m, 4H); 2.63-2.57 (m, 1H); 2.53-2.48 (m, 1H); 1.95-1.85 (m, 3H); 1.58-1.49 (m, 1H), 1.40 (s, 18H); 1.26 (s, 18H). **^{13}C-NMR** ($CDCl_3$) δ ppm: 153.0; 140.6; 136.0; 125.0; 123.4; 121.4; 77.5; 68.3; 57.5; 55,.9; 35.0; 34.1; 31.7; 29.6; 29.6; 25.2. **HRMS** (AuNPET LDI-ToF) m/z: *calculated for* $C_{35}H_{56}NO_3^+$ $[M+H]^+$ = 538.4255 *found* 538.4226, *for* $C_{35}H_{55}NNaO_3^+$ $[M+Na]^+$ = 560.4069 *found* 560.4024. **FT-IR** (ATR diamond, cm^{-1}): 3355.30, 2953.45, 1480.10, 1387.29, 1359.09, 1240.49, 1217.10, 1041.13, 973.63, 880.10, 824.42, 798.87, 758.37, 724.86.

2.2. Synthesis of amine-bis(phenolate) chromium(III) complexes

Chromium(III) complexes **1a-8a** were synthesized in the one-pot two-stage reaction procedure. The ligands were first deprotonated with *n*-butyllithium in THF and then the resulting dilithium salt were reacted with $CrCl_3(THF)_3$ (*Scheme S2*). The applied reaction conditions were selected based on the literature sources.[S3-S8]

The structures of amine-bis(phenolate) chromium(III) complexes **1a-8a** were proved based on the results of HRMS, FT-IR, and ICP-OES analyses. Pink/purple colour observed for complexes **1a-3a, 8a** was indicative of the additional coordination of THF to chromium(III) ions.[S8] However, the HRMS spectra obtained for those complexes comprise only the peaks corresponding to (*ligand*)$CrCl^+$ or (*ligand*)Cr^+ ions without weakly coordinating THF, which is easily detached from the metal ion under MS analysis conditions. Complexes **4a-7a** were green. This colour is typical for the amine-bis(phenolate) chromium(III) complexes without an additional molecule of THF.[S3-S8]

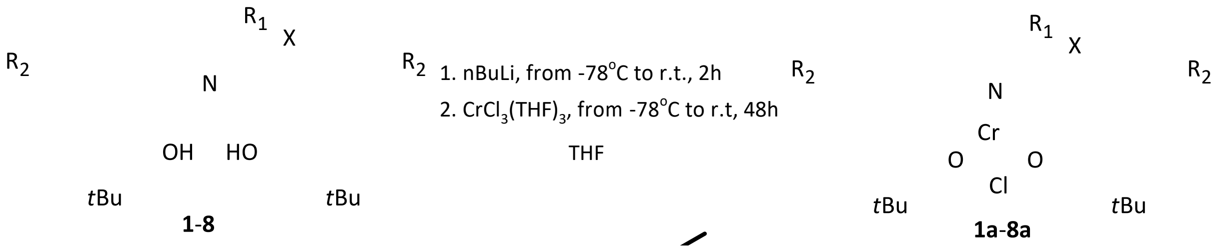

Complex	R_1-X	R_2
1a		*t*Bu
2a	CH_2NMe_2	OMe
3a		F
4a		*t*Bu
5a	CH_2OMe	OMe
6a		F
7a	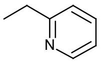	*t*Bu
8a	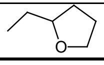	*t*Bu

Scheme S2. Synthesis of amine-bis(phenolate) chromium(III) complexes **1a – 8a**.

General synthesis procedure. The proper amine-bis(phenolate) ligand (1.0 mmol) was transferred to a Schlenk tube under argon atmosphere and 5 mL of anhydrous THF was added. The resulting colourless mixture was cooled to -78°C and 2.5M solution of *n*-butyllithium in hexane was added dropwise (0.9 ml, 2.25 mmol). The cooling bath was removed and then the reaction mixture was stirred at ambient temperature for 2 h. After that time, the colourless or yellow reaction mixture was transferred using a cannula to a suspension of $CrCl_3(THF)_3$ (375 mg, 1 mmol) in 15 mL THF and the resulting mixture was stirred at room temperature for 48 h. During this time, the solid was dissolved and the color of the reaction mixture darkened to purple/dark brown. Finally, the solvent was removed in vacuo and the solid residue was extracted with toluene. The extract was filtered off through celite and toluene was removed in vacuo. The final product was washed with pentane and dried under vacuum for 24 h.

(1a): Yield 0.58, 85%, purple solid. **HRMS** (AuNPET LDI-ToF) m/z: *calculated for* $C_{34}H_{54}ClCrN_2NaO_2^+$ $[M+Na]^+$ = 632.3166 *found* 632.3190, *for* $[M-Cl]^+$ = 574.3579 *found* 574.3619. ***Elemental analysis*** *calculated (%) for* $C_{34}H_{54}ClCrN_2O_2$ (**1a**) Cr 8.52, *for* $C_{38}H_{62}ClCrN_2O_3$ (**1a ·THF**) Cr 7.62 *found* Cr 7.54. **FT-IR** (ATR diamond, cm^{-1}): 3400.28, 2951.28, 1601.96, 1469.14, 1444.18, 1411.88, 1295.45, 1280.50, 1238.08, 1166.24, 1015.34, 875.28, 837.20, 744.15.

(2a): Yield 0.38, 60%, purple solid. **HRMS** (AuNPET LDI-ToF) m/z: *calculated for* $C_{28}H_{42}ClCrN_2NaO_4^+$ $[M+Na]^+$ = 580.2125 *found* 580.1933, *for* $[M-Cl]^+$ = 522.2544 *found* 522.2385. ***Elemental analysis*** *calculated (%) for* $C_{28}H_{42}ClCrN_2O_4$ (**2a**) Cr 9.32, *for* $C_{32}H_{50}ClCrN_2O_5$ (**2a ·THF**) Cr 8.25 *found* Cr 7.95. **FT-IR** (ATR diamond, cm^{-1}): 3364.17, 2952.72, 1602.06, 1467.80, 1444.18, 1360.53, 1295.69, 1256.16, 1237.59, 1202.64, 1166.24, 1014.86, 837.68, 804.17.

(3a): Yield 0.54g, 89%, pink solid. **HRMS** (AuNPET LDI-ToF) m/z: *calculated for* $C_{26}H_{36}ClCrF_2N_2NaO_4^+$ $[M+Na]^+$ = 556.1725 *found* 556.1661, *for* $[M-Cl]^+$ = 498.2139 *found* 498.2038. ***Elemental analysis*** *calculated (%) for* $C_{26}H_{36}ClCrF_2N_2O_4$ (**3a**) Cr 9.74, *for* $C_{30}H_{44}ClCrF_2N_2O_5$ (**3a ·THF**) Cr 8.58 *found* Cr 8.67. **FT-IR** (ATR diamond, cm^{-1}): 2961.40, 2870.28, 1604.00, 1469.25, 1432.61, 1292.80, 1260.25, 1200.71, 1095.61, 1018.23, 936.27, 867.57, 794.29, 773.32.

(4a): Yield 0.24 g, 40%, green solid. **HRMS** (AuNPET LDI-ToF) m/z: *calculated for* $C_{33}H_{51}ClCrNNaO_3^+$ $[M+Na]^+$ = 619.2849 *found* 619.2779; *for* $[M-Cl]^+$ = 561.3263 *found* 561.3219. ***Elemental analysis*** *calculated (%) for* $C_{33}H_{51}ClCrNO_3$ (**4a**) Cr 8.71 *found* Cr 8.40. **FT-IR** (ATR diamond, cm^{-1}): 3366.86, 2952.00, 1603.04, 1475.61, 1438.88, 1360.53, 1259.77, 1202.40, 1094.65, 1012.20, 801.76,

(5a): Yield 0.23 g, 42%, green solid. **HRMS** (AuNPET LDI-ToF) m/z: *calculated for* $C_{27}H_{39}ClCrNNaO_5^+$ $[M+Na]^+$ = 567.1809 *found* 567.1749; *for* $[M-Cl]^+$ = 509.2222 *found* 509.2152. ***Elemental analysis*** *calculated (%) for* $C_{27}H_{39}ClCrNO_5$ (**5a**) Cr 9.54 *found* Cr 9.19 **FT-IR** (ATR diamond, cm^{-1}): 3372.02, 2956.58, 1603.31, 1463.95, 1422.97, 1318.35, 1240.73, 1206.01, 1093.44, 1058.01, 1014.37, 804.41, 792.12.

(6a): Yield 0.284 g, 55%, green solid. **HRMS** (AuNPET LDI-ToF) m/z: *calculated for* $C_{25}H_{33}ClCrF_2NNaO_3^+$ $[M+Na]^+$ = 543.1414 *found* 543.1384; *for* $[M-Cl]^+$ = 485.1828 *found* 485.1737. ***Elemental analysis*** *calculated (%) for* $C_{25}H_{33}ClCrF_2NO_3$ (**6a**) Cr 9.98 *found* Cr 9.61. **FT-IR** (ATR diamond, cm^{-1}): 3143.16, 2957.30, 1615.09, 1539.40, 1421.04, 1362.94, 1421.04, 1362.94, 1259.53, 1199.27, 1095.37, 1012.93, 863.23, 794.29, 728.24.

(7a): Yield 0.60 g, 95%, dark green/brown solid. **HRMS** (AuNPET LDI-ToF) m/z: *calculated for* $C_{36}H_{50}ClCrN_2NaO_2$ $[M+Na]^+$ = 652.2858 *found* 652.2805; *for* $[M-Cl]^+$ = 594.3272 *found* 594.3255. ***Elemental analysis*** *calculated (%) for* $C_{36}H_{50}ClCrN_2O_2$ (**7a**) Cr 8.25 *found* Cr 7.99. **FT-IR** (ATR diamond, cm^{-1}): 2950.55, 1611.48, 1597.98, 1570.98, 1478.41, 1435.98, 1360.53, 1260.98, 1236.87, 1166.72, 1094.16, 1003.53, 800.31, 756.92, 728.96.

(8a): Yield 0.45 g, 72%, purple solid. **HRMS** (AuNPET LDI-ToF) m/z: *calculated for* $C_{35}H_{53}ClCrNNaO_3$ $[M+Na]^+$ = 645.3006 *found* 645.2972; *for* $[M-Cl]^+$ = 587.3425 *found* 587.3407. ***Elemental analysis*** *calculated (%) for* $C_{35}H_{53}ClCrNO_3$ (**8a**) Cr 8.34, *for* $C_{39}H_{61}ClCrNO_4$ (**8a·THF**) Cr 7.48 *found* Cr 7.19. **FT-IR** (KBr, cm^1): 3390.49, 2952.48,

1633.17, 1603.04, 1477.69, 1439.60, 1360.53, 1259.77, 1167.45, 1081.39, 1012.20, 799.59, 746.80,

3. Spectra

3.1. ¹H-NMR spectra of the obtained polymer

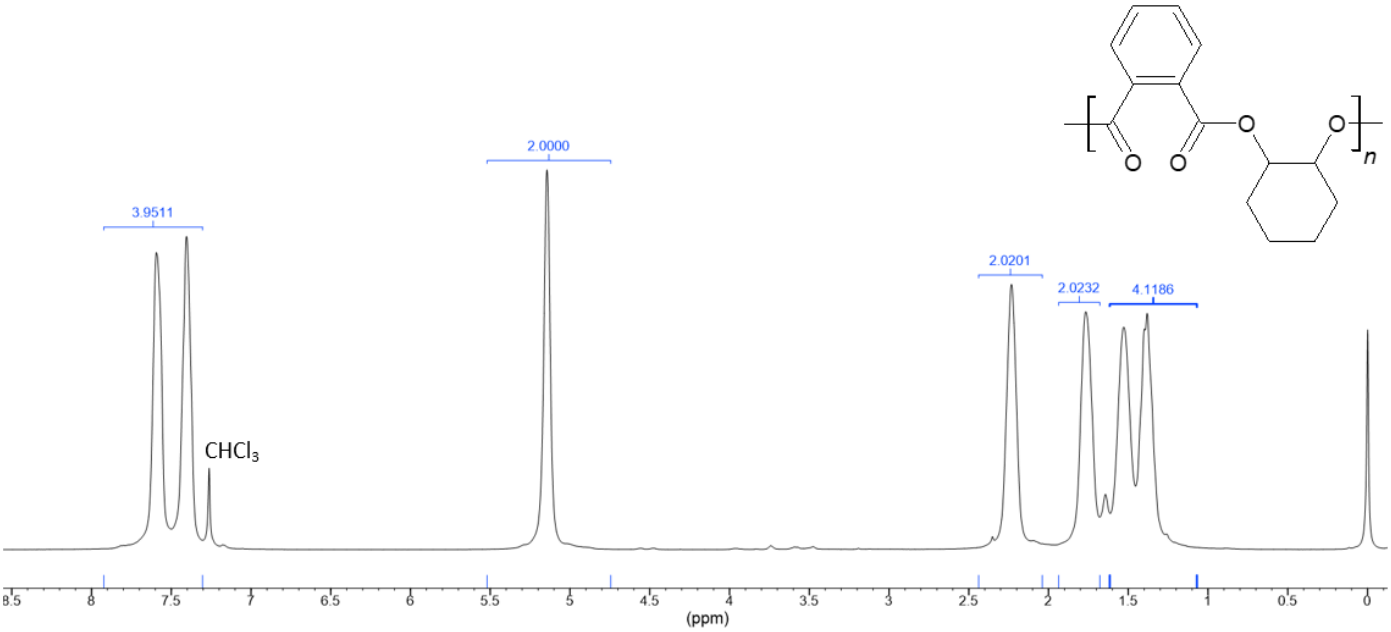

Figure S1. ¹H-NMR (CDCl₃) spectrum of the obtained poly(cyclohexene phthalate) (Table 4 Run 2).

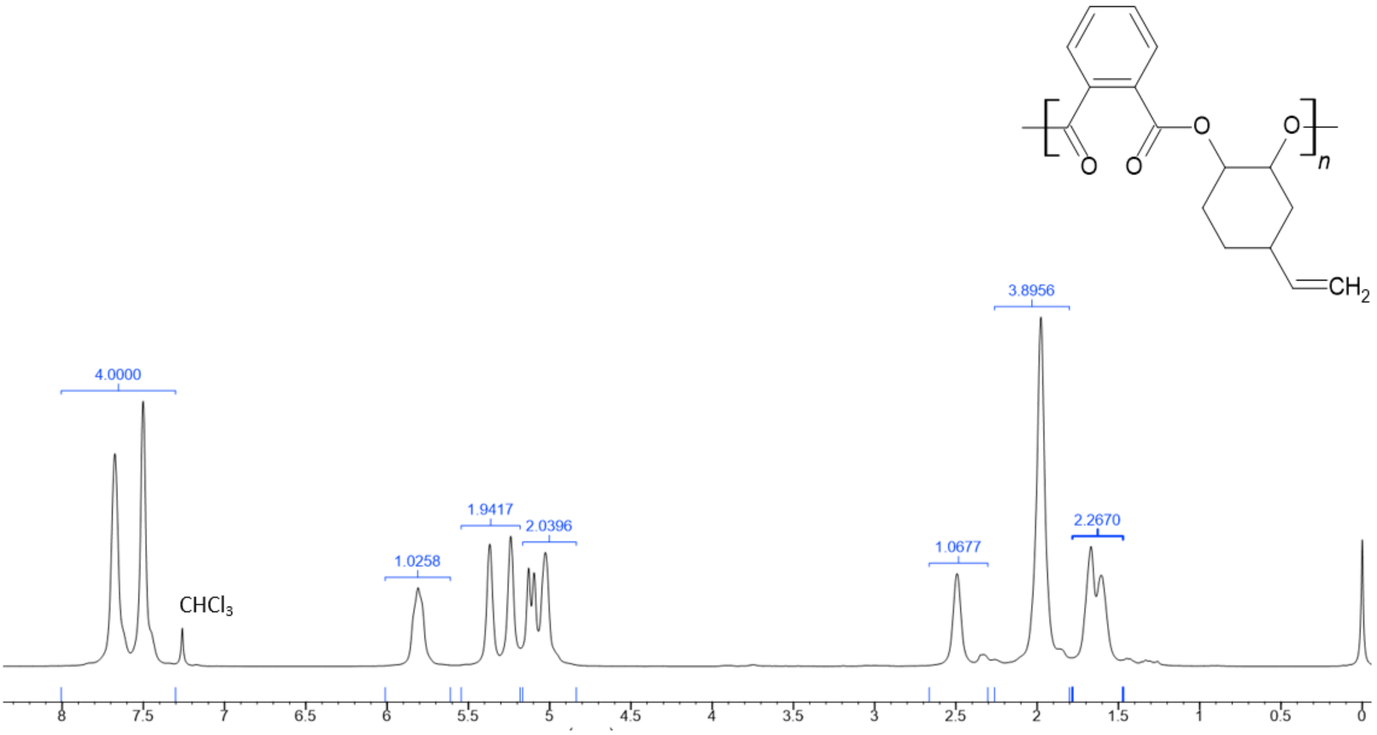

Figure S2. ¹H-NMR (CDCl₃) spectrum of the obtained poly(4-vinylcyclohexen phthalate) (Table 4 Run 4).

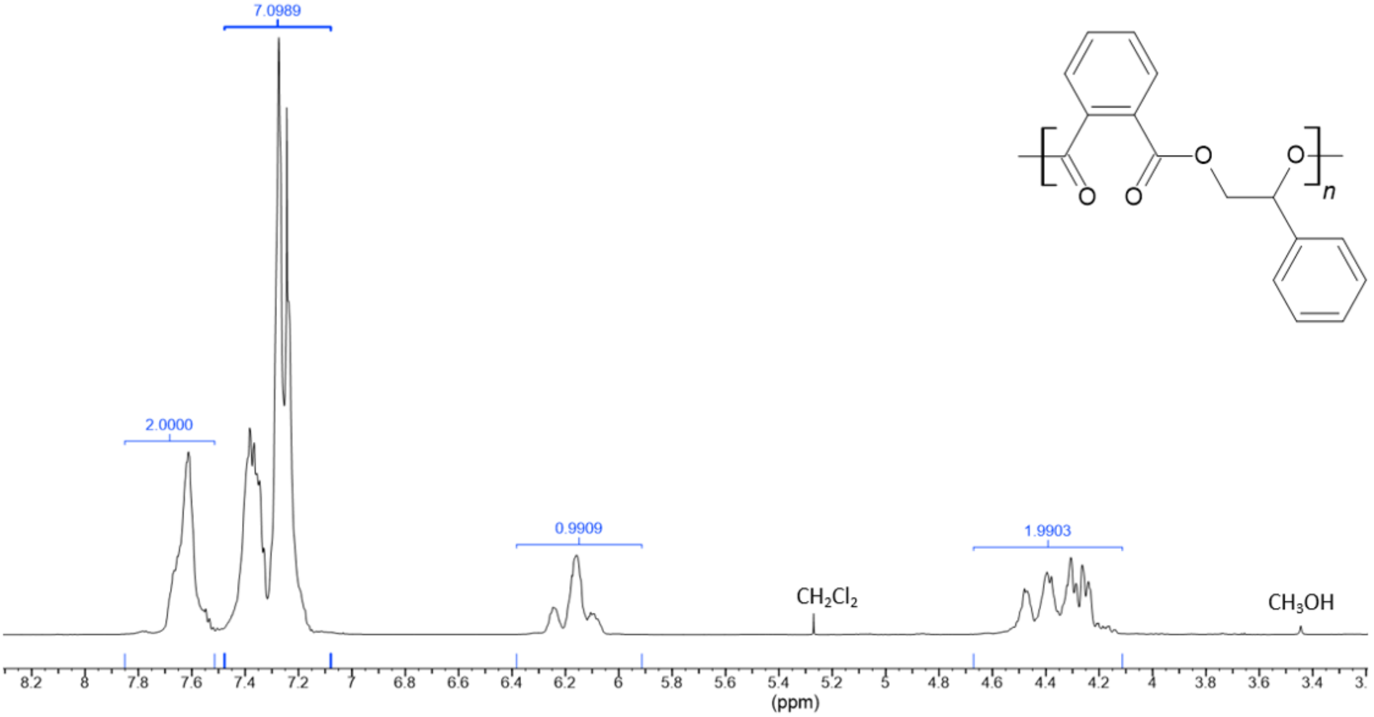

Figure S3. ^{1}H-NMR ($CDCl_3$) spectrum of the obtained poly(phenylethylene phthalate) (Table 4 Run 6).

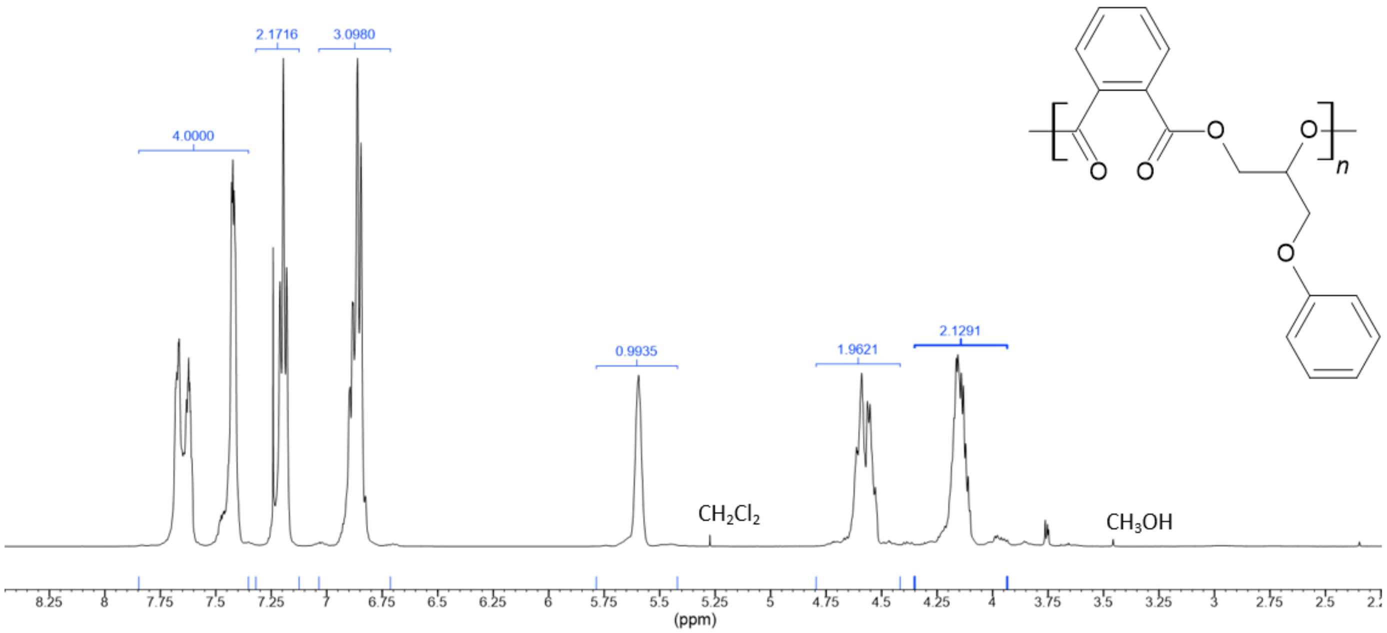

Figure S4. ^{1}H-NMR ($CDCl_3$) spectrum of the obtained poly(phenoxymethylethylene phthalate) (Table 4 Run 8).

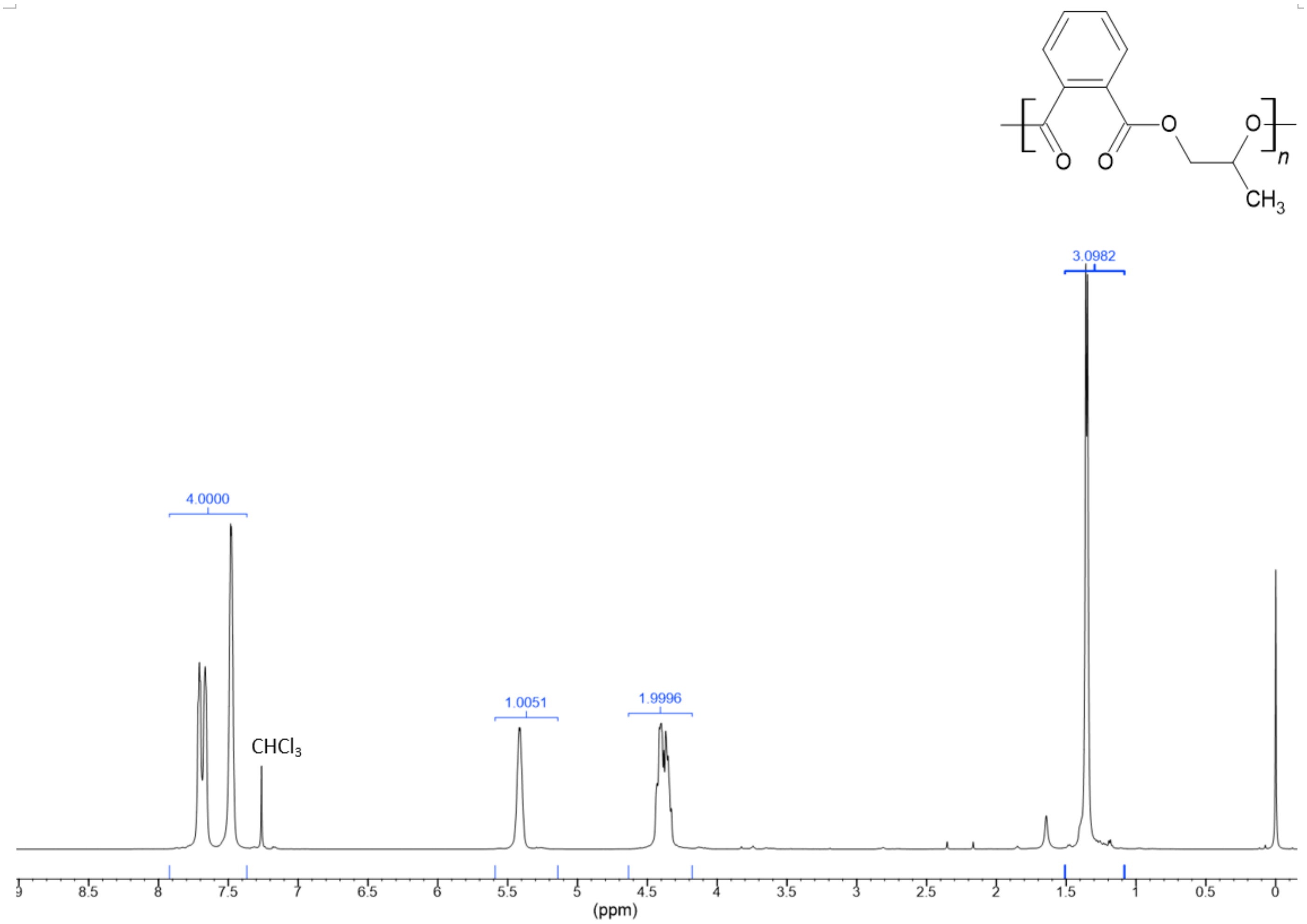

Figure S5. ^{1}H-NMR ($CDCl_3$) spectrum of the obtained poly(propylene phthalate) (Table 4 Run 10).

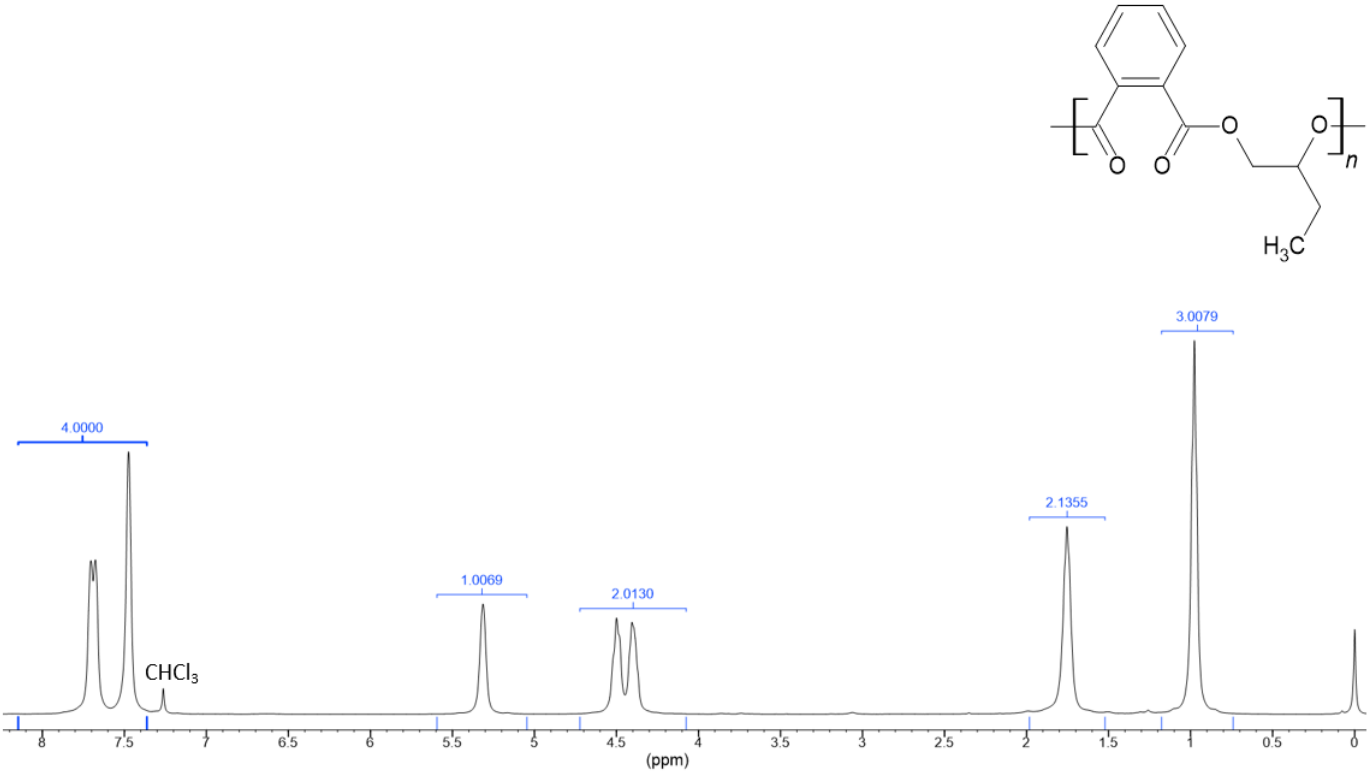

Figure S6. ^{1}H-NMR ($CDCl_3$) spectrum of the obtained poly(butylene phthalate) (Table 4 Run 12).

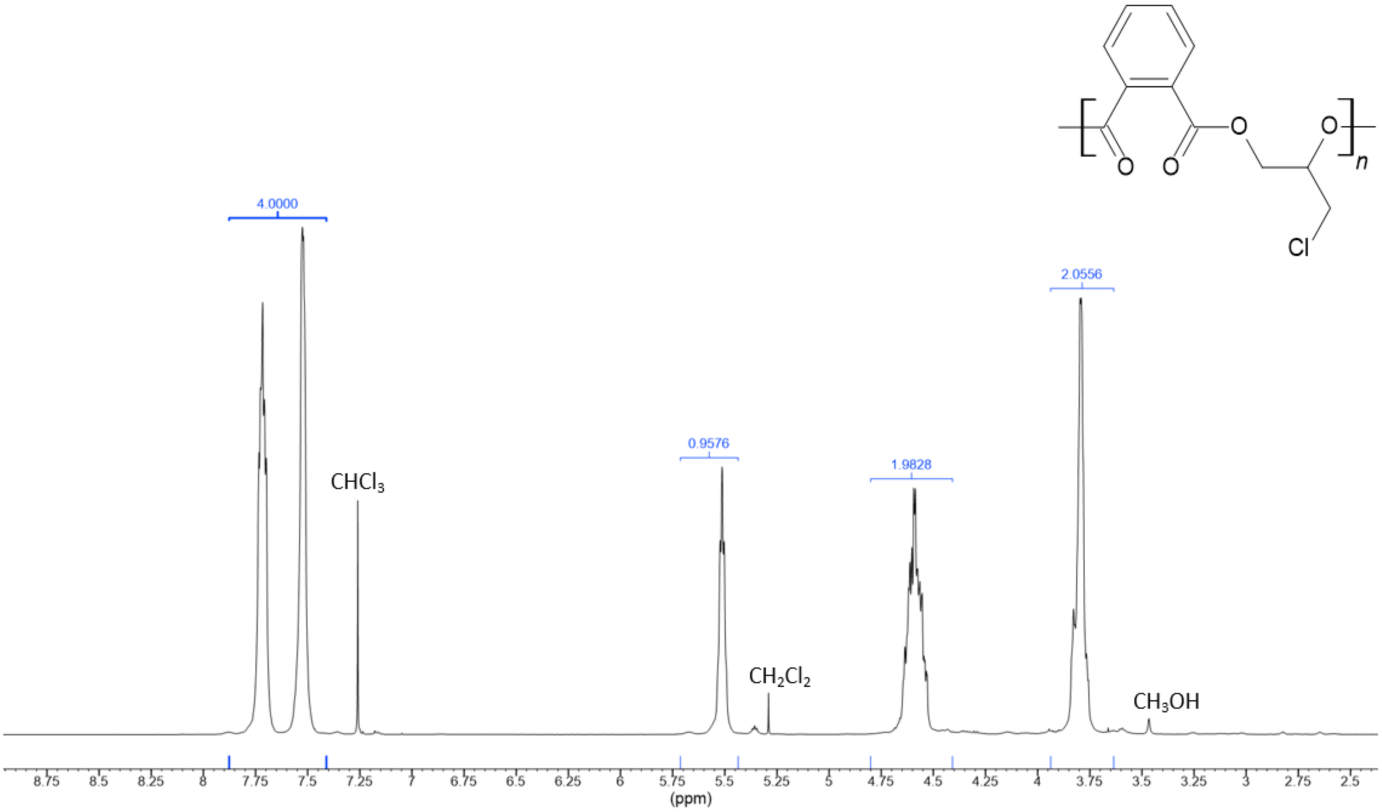

Figure S7. ^{1}H-NMR ($CDCl_3$) spectrum of the obtained poly(chloromethylethylene phthalate) (Table 4 Run 14).

3.2. FTIR spectra of the obtained polyesters

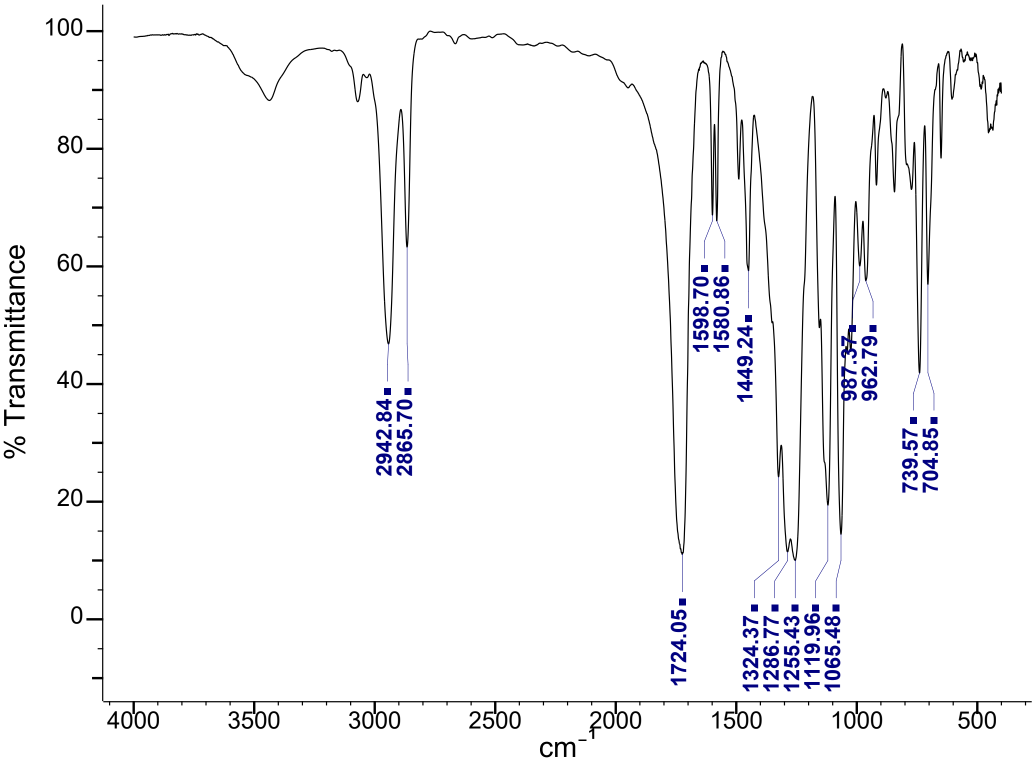

Figure S8. FT-IR (KBr) spectrum of the obtained poly(cyclohexene phthalate) (Table 4 Run 2).

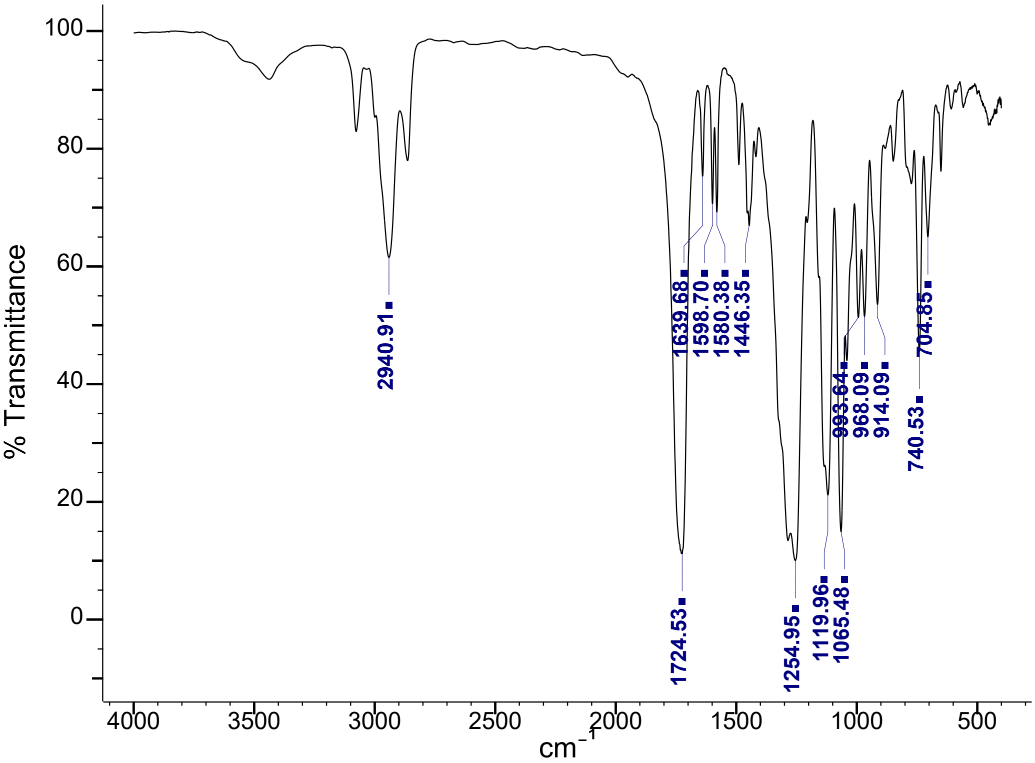

Figure S9. FT-IR (KBr) spectrum of the obtained poly(4-vinylcyclohexene phthalate) (Table 4 Run 4).

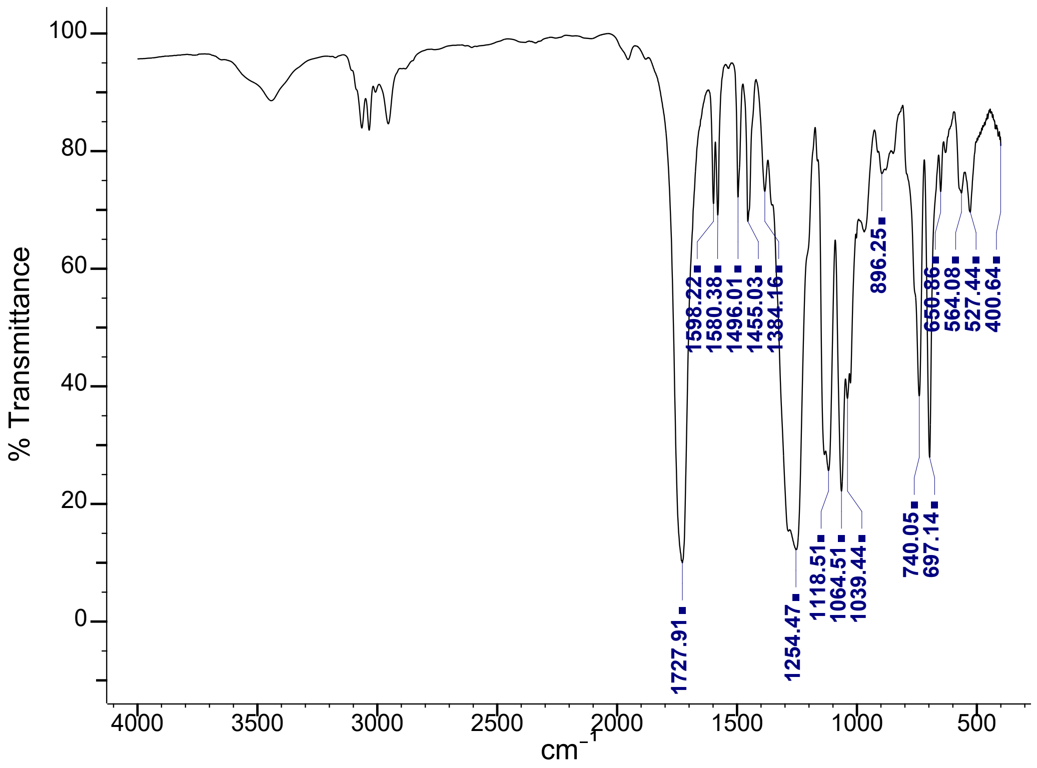

Figure S10. FT-IR (KBr) spectrum of the obtained poly(phenylethylene phthalate) (Table 4 Run 6).

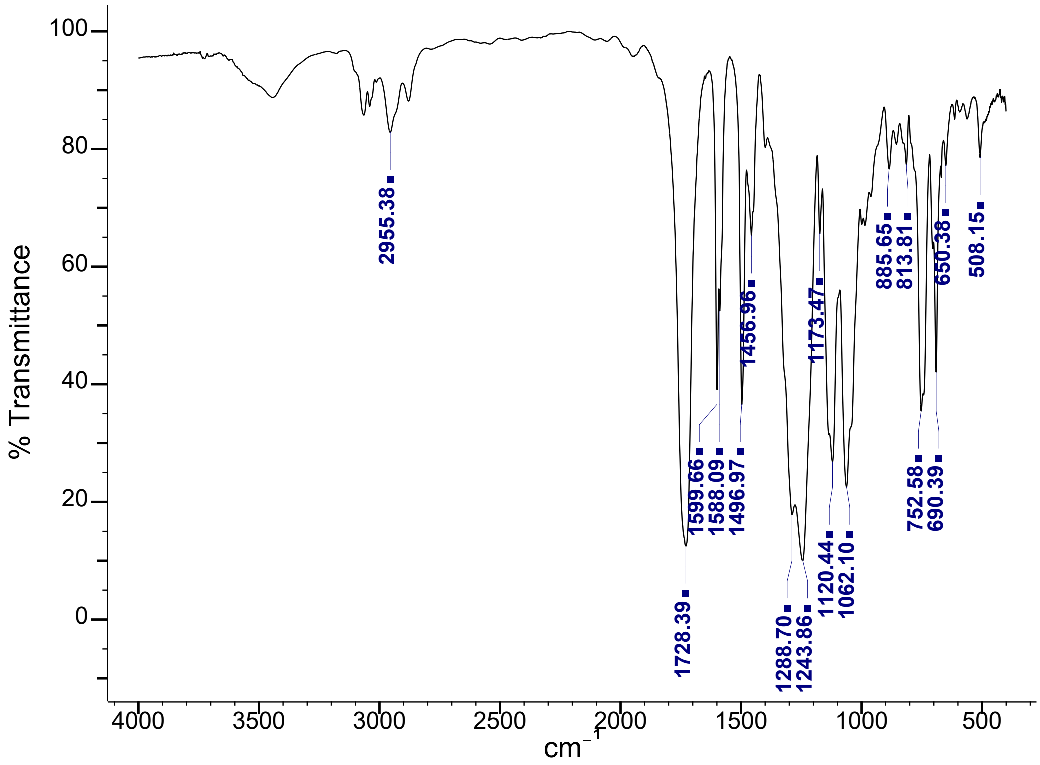

Figure S11. FT-IR (KBr) spectrum of the obtained poly(phenoxymethylethylene phthalate) (Table 4 Run 8).

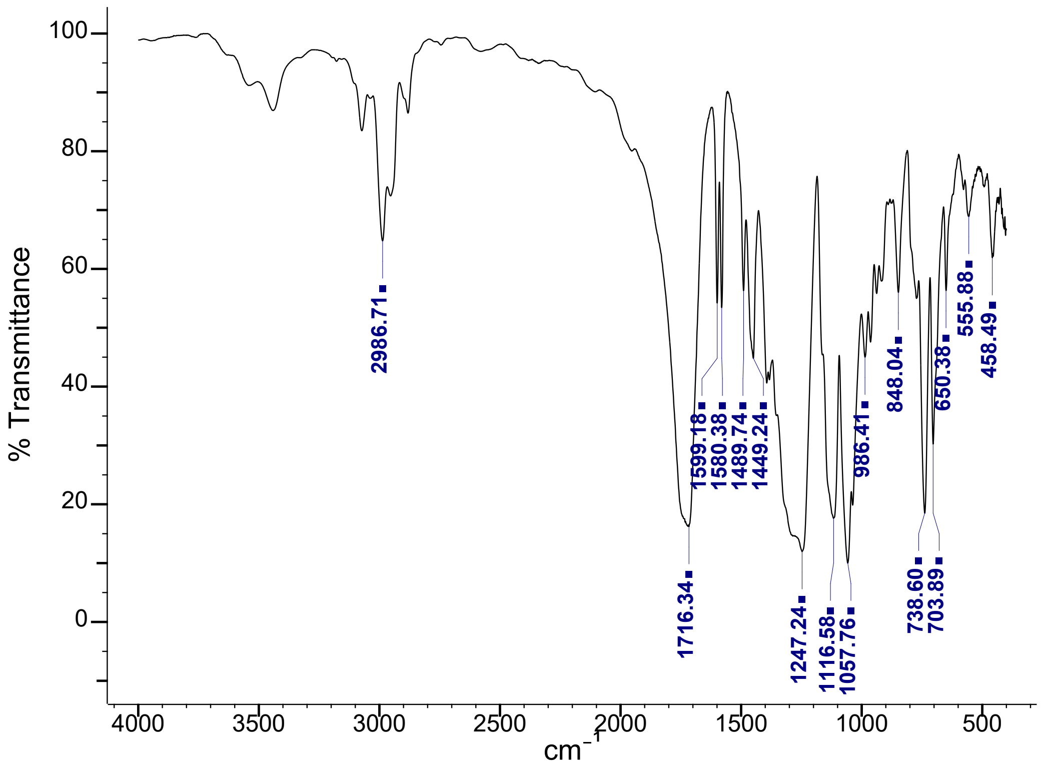

Figure S12. FT-IR (KBr) spectrum of the obtained poly(propylene phthalate) (Table 4 Run 10).

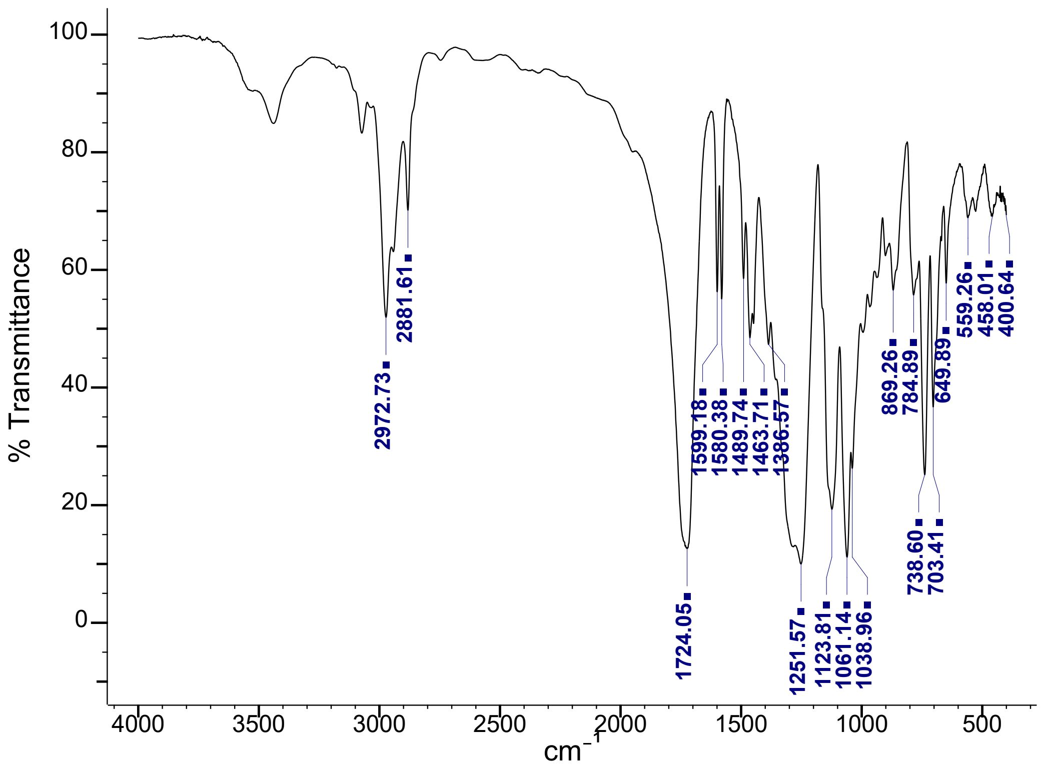

Figure S13. FT-IR (KBr) spectrum of the obtained poly(buthylene phthalate) (Table 4 Run 12).

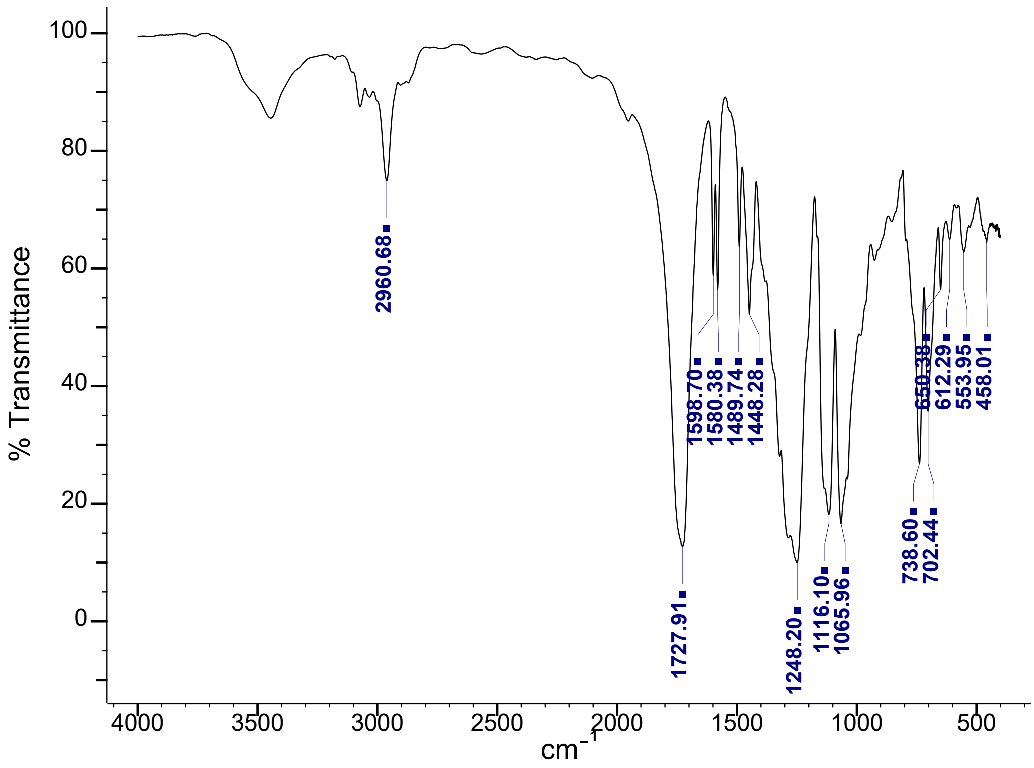

Figure S14. FT-IR (KBr) spectrum of the obtained poly(chloromethylethylene phthalate) (Table 4 Run 14).

4. DSC thermograms

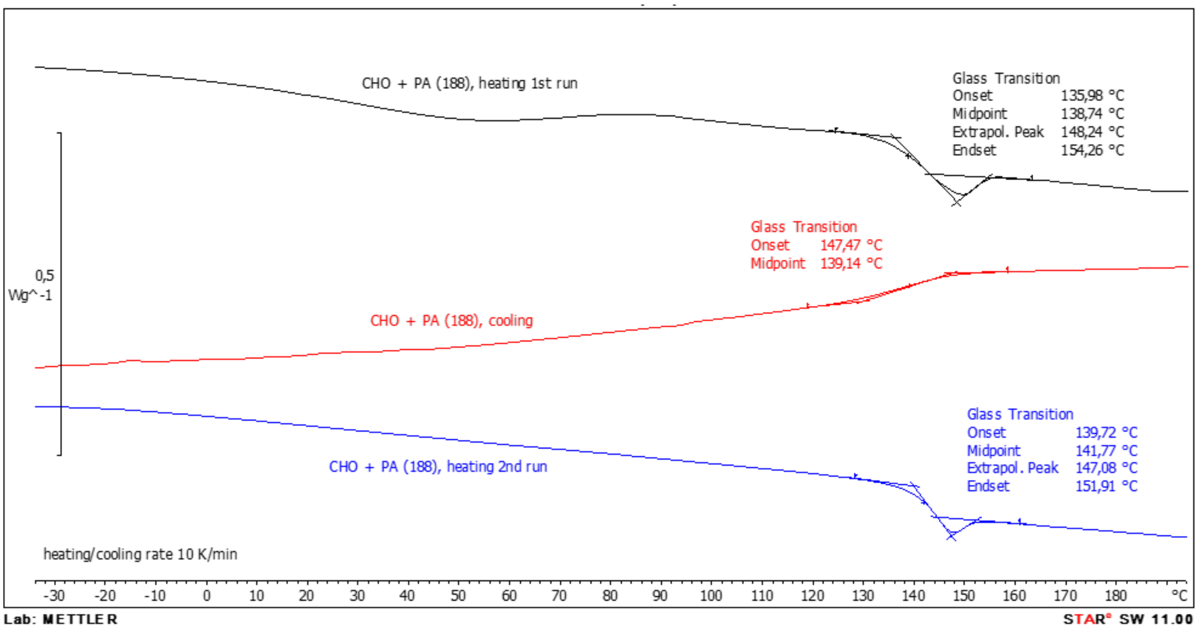

Figure S15. DSC thermograms of the obtained poly(cyclohexene phthalate) (Table 4 Run 2).

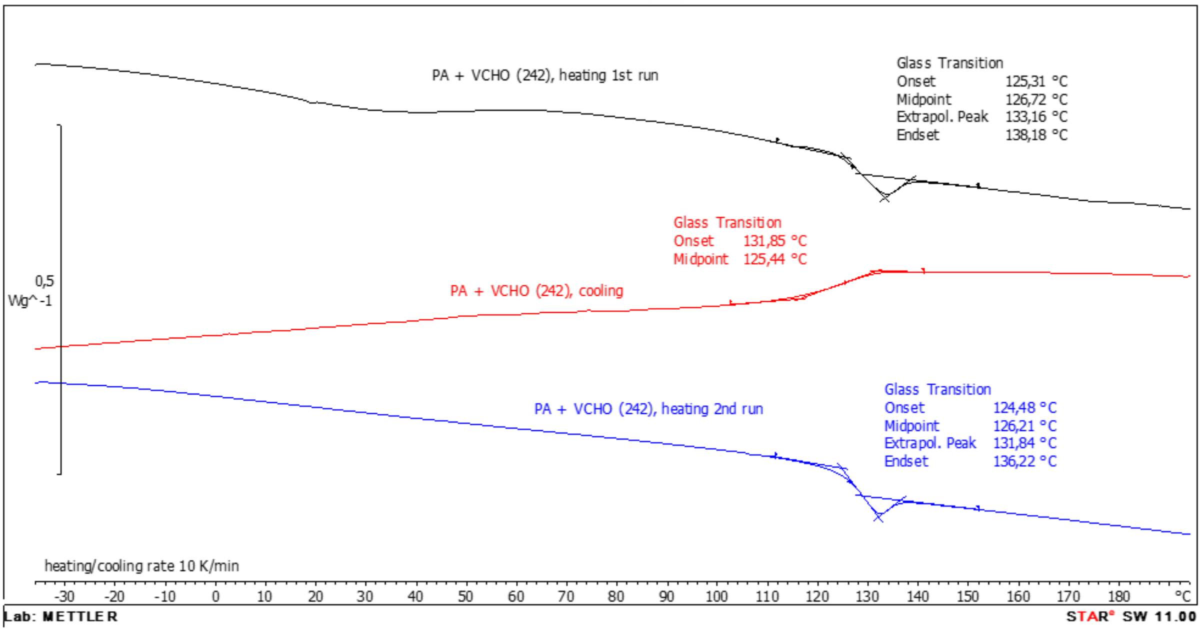

Figure S16. DSC thermograms of the obtained poly(4-vinylcyclohexene phthalate) (Table 4 Run 4).

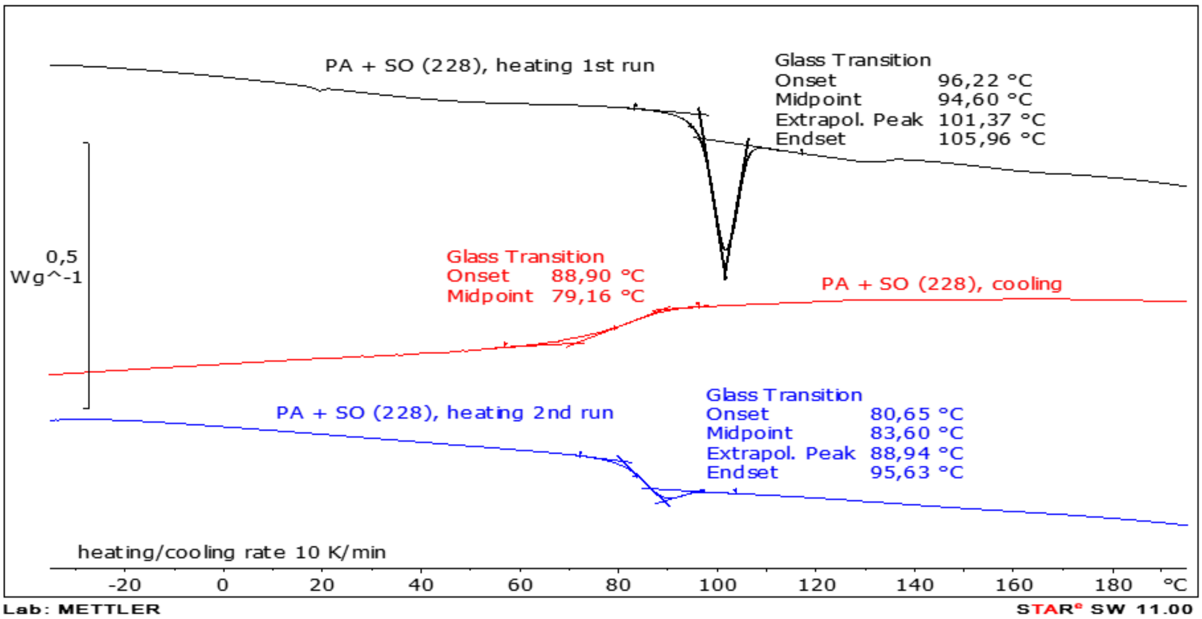

Figure S17. DSC thermograms of the obtained poly(phenylethylene phthalate) (Table 4 Run 6).

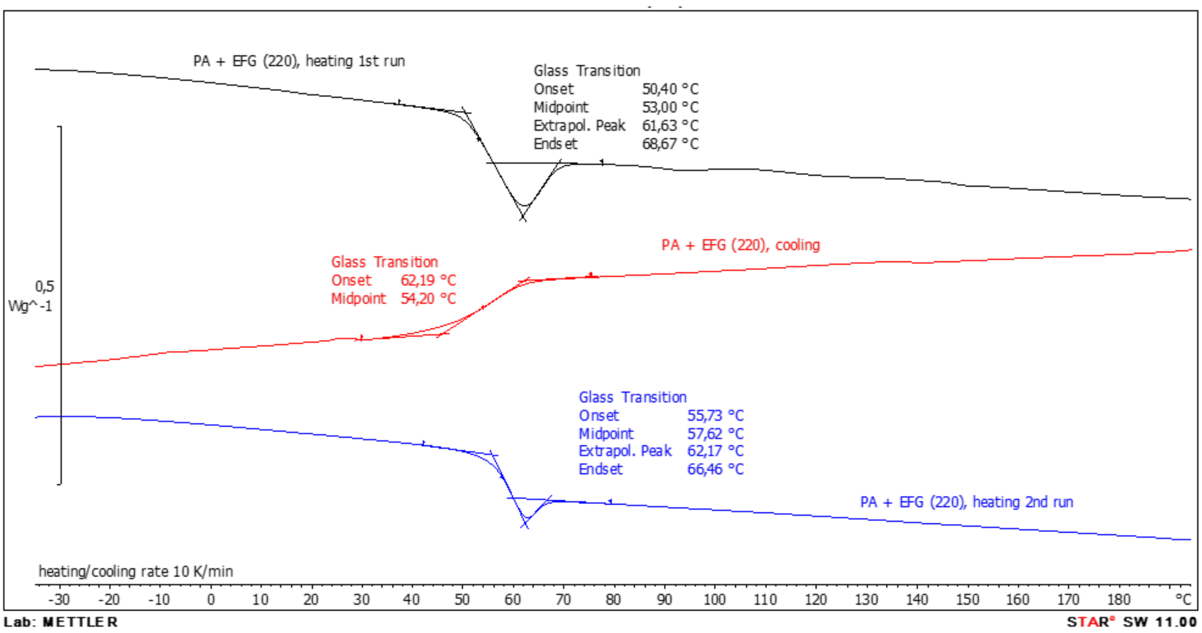

Figure S18. DSC thermograms of of the obtained poly(phenoxymethylethylene phthalate) (Table 4 Run 8).

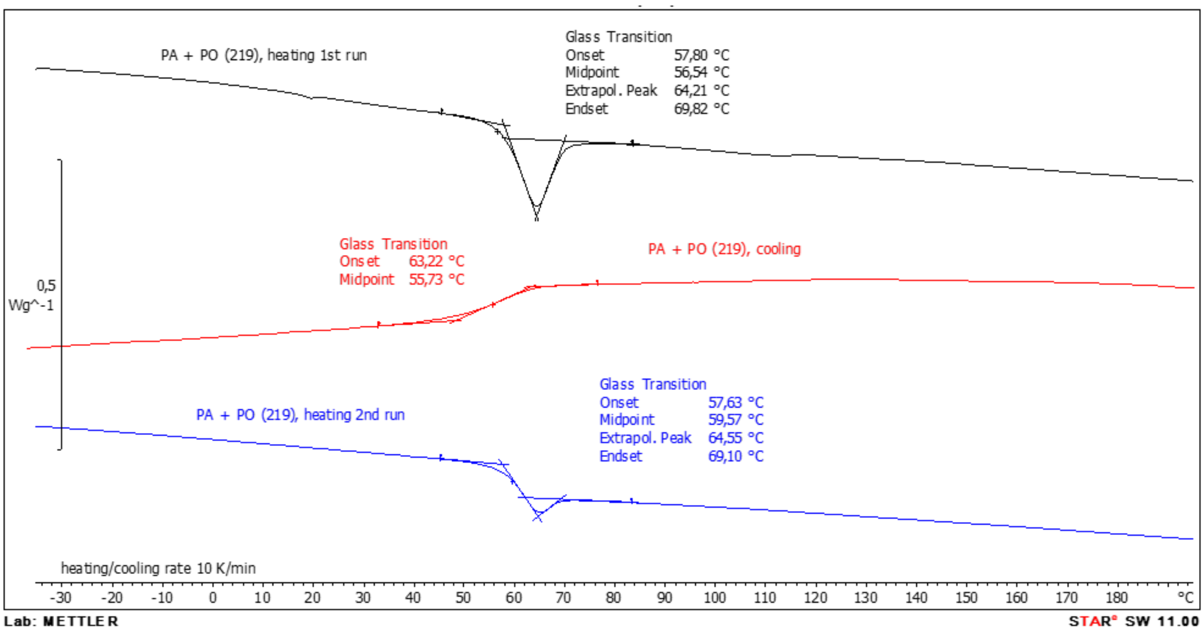

Figure S19. DSC thermograms of the obtained poly(propylene phthalate) (Table 4 Run 10).

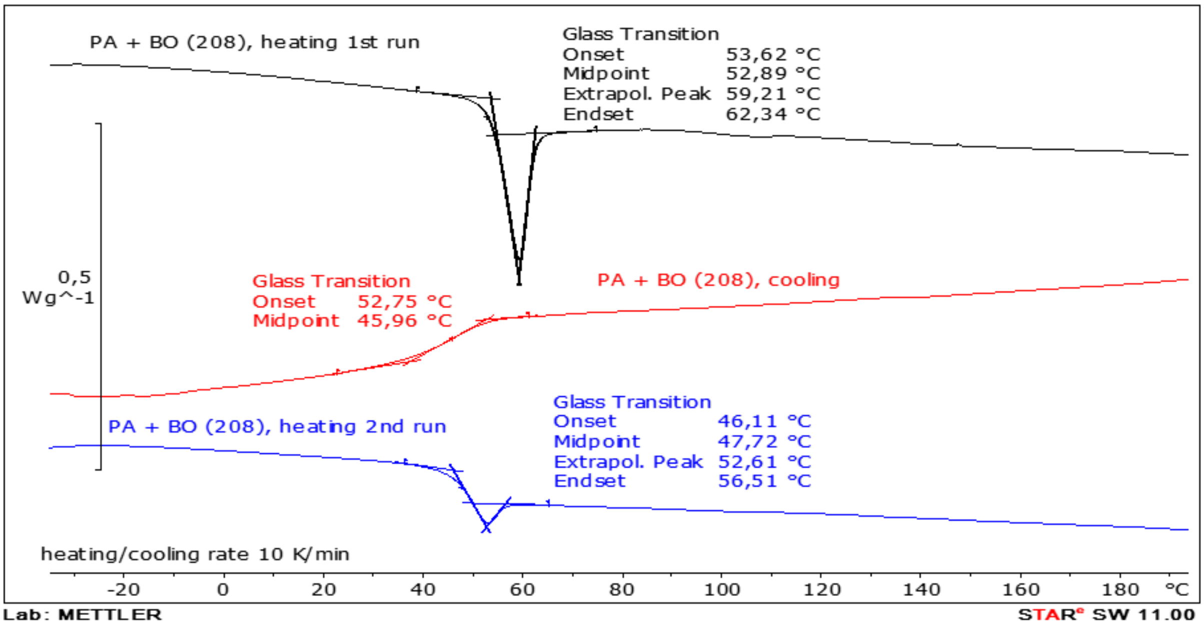

Figure S20. DSC thermogram of the obtained poly(buthylene phthalate) (Table 4 Run 12).

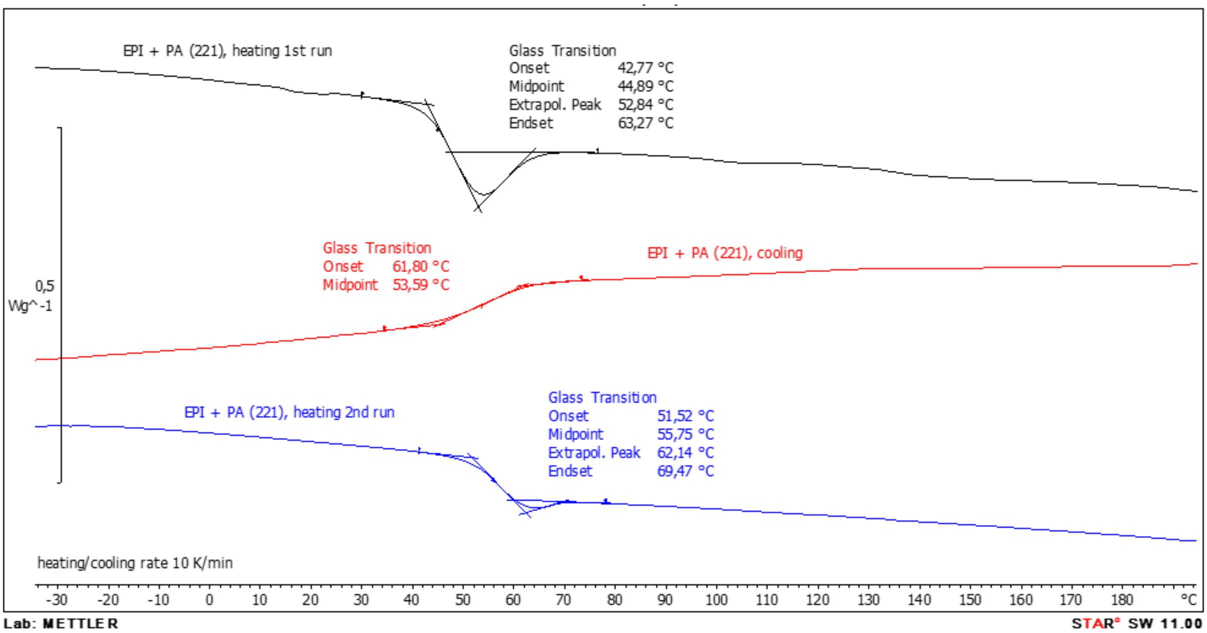

Figure S21. DSC thermogram of the obtained poly(chloromethylethylene phthalate) (Table 4 Run 14).

5. Representative GPC traces

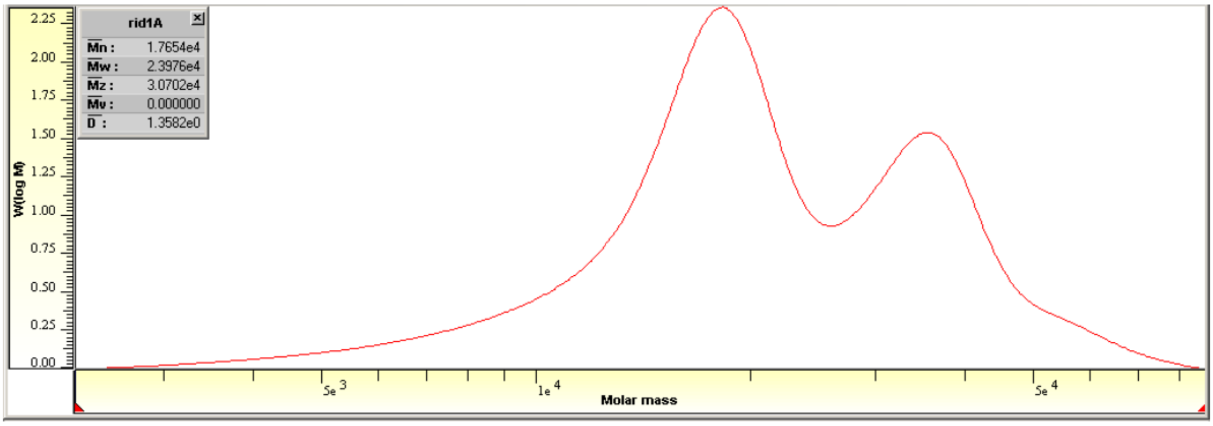

Figure S22. GPC trace of the obtained poly(cyclohexene phthalate) with M_n = 17.6 kg/mol, Đ = 1.36 (Table 4 Run 2).

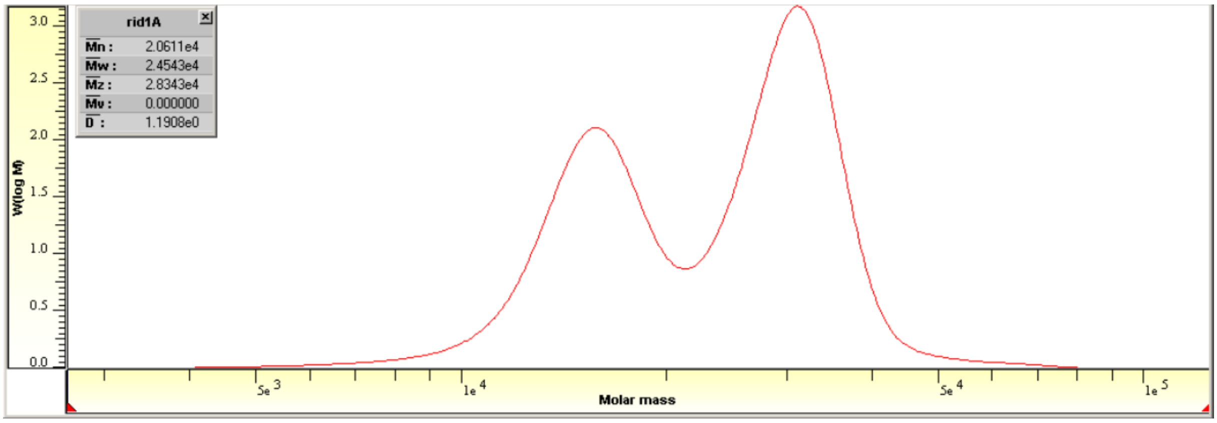

Figure S23. GPC trace of the obtained poly(4-vinyl cyclohexene phthalate) with M_n = 20.6 kg/mol, Đ = 1.19 (Table 4 Run 4).

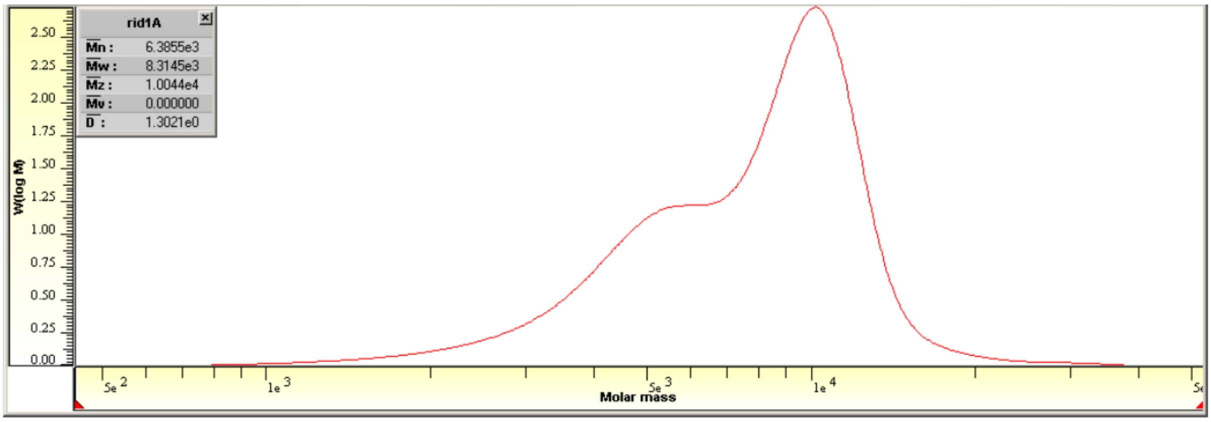

Figure S24. GPC trace of the obtained poly(phenylethylene phthalate) with M_n = 6.4 kg/mol, mol, Đ = 1.30 (Table 4 Run 6).

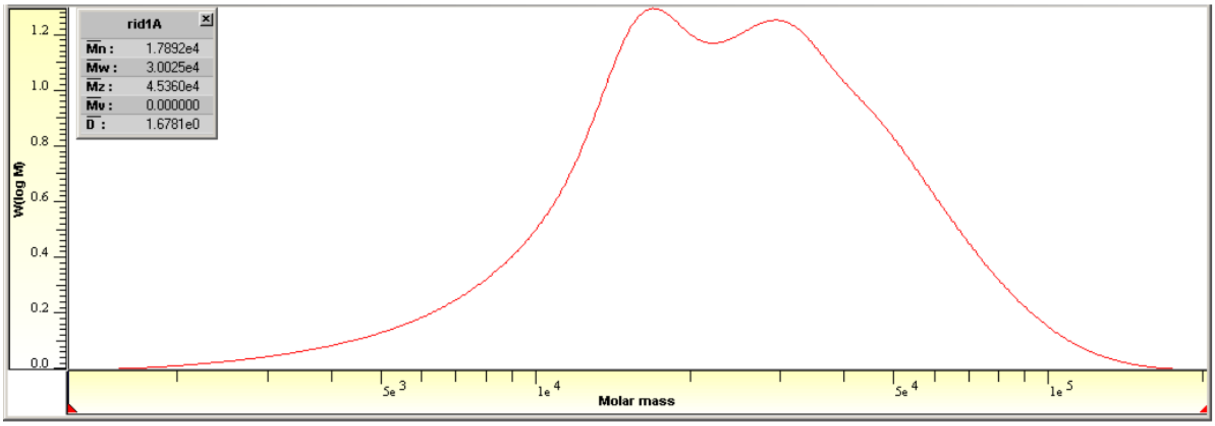

Figure S25. GPC trace of the obtained poly(phenoxymethylethylene phthalate) with M_n = 17.9 kg/mol, Đ = 1.68 (Table 4 Run 8).

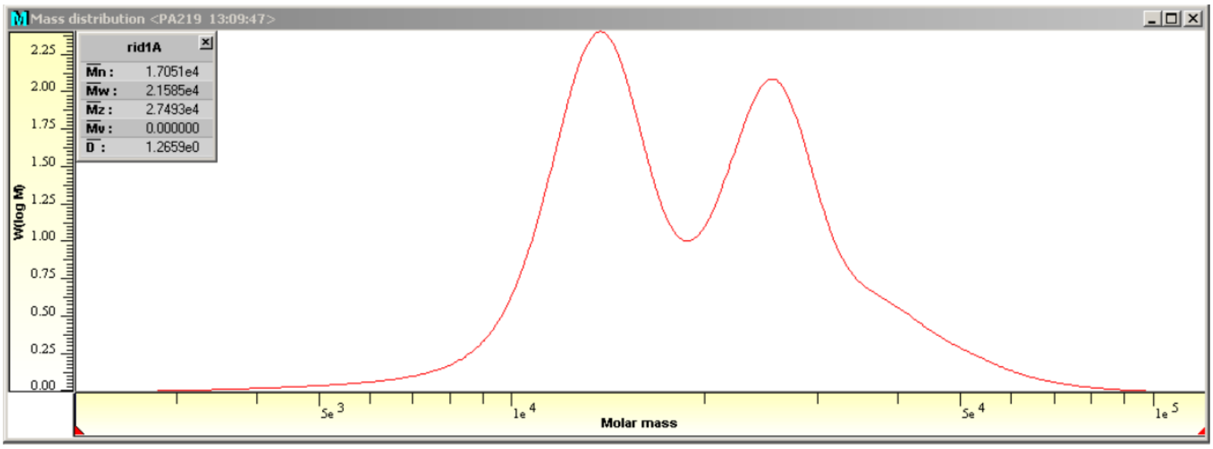

Figure S26. GPC trace of the obtained poly(propylene phthalate) with M_n = 17.0 kg/mol, Đ = 1.27 (Table 4 Run 10).

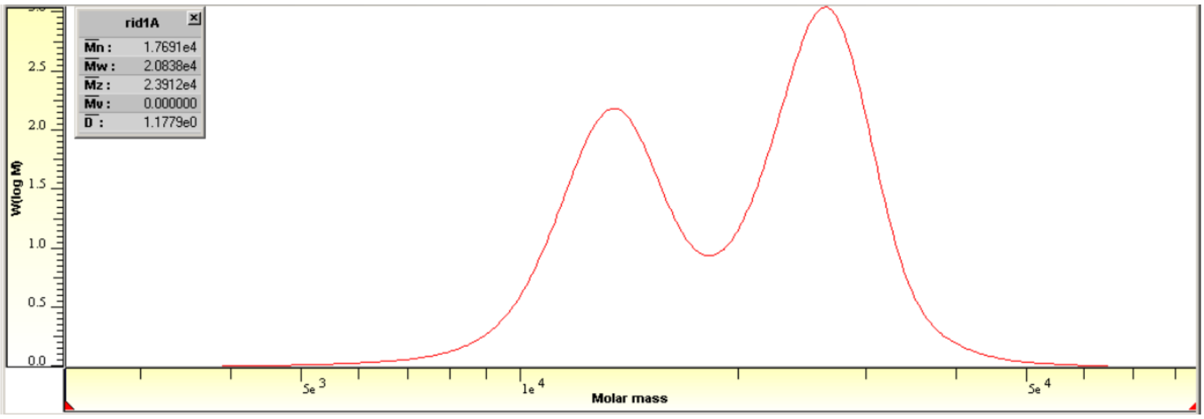

Figure S27. GPC trace of the obtained poly(butylene phthalate) with M_n = 17.7 kg/mol, M_w = 18.5 g/mol, Đ = 1.18 (Table 4 Run 12).

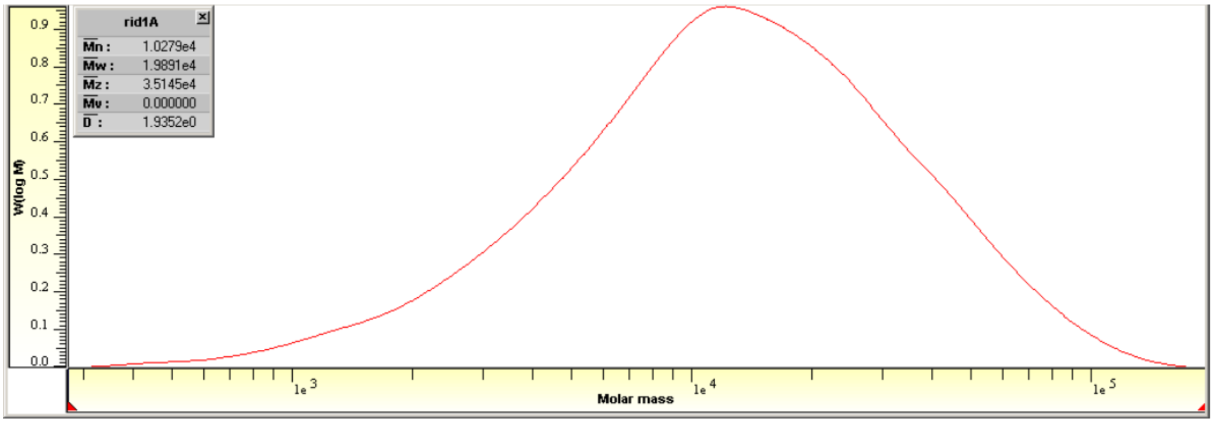

Figure S28. GPC trace of the obtained poly(chloromethylethylene phthalate) with M_n = 10.3 kg/mol, Đ = 1.93. (reaction time 240 min) (Table 4 Run 14).

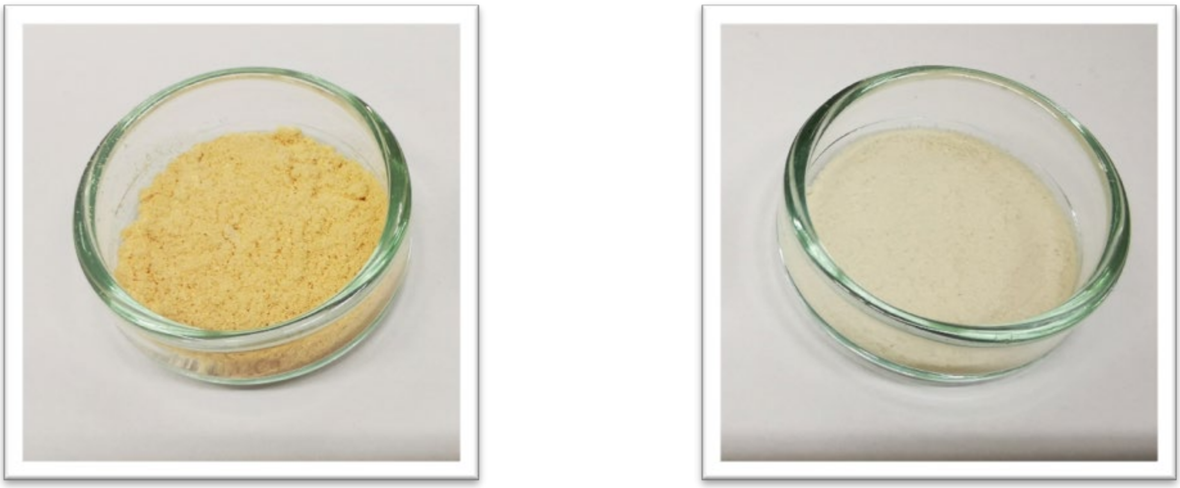

Figure S29. Photographs of the isolated poly(cyclohexylene phtalates): (a) obtained using (salophen)CrCl/DMAP as catalyst (after three-time precipitation with methanol), (b) obtained using complex **3a**/DMAP as catalyst (after single precipitation with methanol).

References:

S1 DiCiccio, A.M.; Longo, J.M.; Rodríguez-Calero, G.G.; Coates, G.W. Development of Highly Active and Regioselective Catalysts for the Copolymerization of Epoxides with Cyclic Anhydrides: An Unanticipated Effect of Electronic Variation, *J. Am. Chem. Soc*. **2016**, *138*, 7107 (Supporting information).

S2 Sekuła, J.; Nizioł, J.; Rode, W.; Ruman, T. Gold nanoparticle-enhanced target (AuNPET) as universal solution for laser desorption/ionization mass spectrometry analysis and imaging of low molecular weight compounds, *Anal. Chim. Acta* **2015**, *875*, 61.

S3 Dean, R.K.; Granville, S.L.; Dawe, L.N.; Decken, A.; Hattenhauera, K.M.; Kozak, C.M. Structure and magnetic behaviour of mono- and bimetallic chromium(iii) complexes of amine-bis(phenolate) ligands, *Dalton Trans*., **2010**, *39*, 548.

S4 Dean, R.K.; Dawe, L.N.; Kozak, C.M. Copolymerization of Cyclohexene Oxide and CO2 with a Chromium Diamine-bis(phenolate) Catalyst, *Inorg. Chem*. **2012**, *51*, 9095.

S5 Dean, R.K.; Devaine-Pressing, K.; Dawe, L.N. and Kozak, C.M. Reaction of CO2 with propylene oxide and styrene oxide catalyzed by a chromium(iii) amine-bis(phenolate) complex, *Dalton Trans*. **2013**, *42*, 9233.

S6 Devaine-Pressing, K.; Dawe, L.N.; Kozak, C.M. Cyclohexene oxide/carbon dioxide copolymerization by chromium(III) amino-bis(phenolato) complexes and MALDI-TOF MS analysis of the polycarbonates, *Polym. Chem*. **2015**, *6*, 6305 (Supporting information).

S7 Ni, K.; Kozak, C.M. Kinetic Studies of Copolymerization of Cyclohexene Oxide with CO2 by a Diamino-bis(phenolate) Chromium(III) Complex, *Inorg. Chem*., **2018**, *57*, 3097.

S8 Ni, K.; Paniez-Grave, V.; Kozak, C. M. Effect of Azide and Chloride Binding to Diamino-bis(phenolate) Chromium Complexes on CO2/Cyclohexene Oxide Copolymerization, *Organometallics* **2018**, *37*, 2507.
